# The role of cervical microbiome in cervical incompetence: insights from 16 S rRNA metagenomic sequencing

**DOI:** 10.1186/s12866-025-04203-0

**Published:** 2025-08-06

**Authors:** Jiang Jingwen, Fu Jingran, Miao Liye, Han Yu, Meng Yancen, Zhang Jingya

**Affiliations:** Department of Obstetrics and Gynecology, Shijiazhuang Fourth Hospital, Shijiazhuang, 050000 Hebei China

**Keywords:** Cervical incompetence, Preterm birth, Cervical microbiome, TLR/NF-κB signaling pathway

## Abstract

Cervical incompetence (CI) is recognized as a critical factor contributing to mid-pregnancy miscarriage and preterm delivery, significantly affecting pregnancy outcomes. Despite this, the specific role of the microbiome in this pathological process remains inadequately understood. This study seeks to elucidate the core microbiome associated with CI in pregnant women and explore its potential biological mechanisms. Utilizing 16 S rRNA metagenomic sequencing, we examined the cervical mucus microbiota of women with CI both pre-operatively (PreOp) and post-operatively (PostOp). Subsequently, the immunomodulatory effects of these microbial communities on the immune system were systematically assessed using quantitative real-time PCR, Western blotting, and enzyme-linked immunosorbent assay. The findings revealed a significant reduction in microbial diversity and richness in PostOp cervical mucus, alongside notable alterations in microbiota composition. The genera *Lactobacillus*, *Bifidobacterium*, *Gardnerella*, *Streptococcus*, and *Anaerococcus* were identified as predominant. Further analysis demonstrated that treatment with 25% *Lactobacillus crispatus* (*L. crispatus*) supernatants, in comparison to 25% Group B Streptococcus (GBS) supernatants, resulted in high cell viability and normal morphology in HcerEpic cells. Importantly, the combination of 25% *L. crispatus* and 25% GBS supernatants significantly reduced the mRNA and protein expression levels of Toll-like receptor 4 (TLR4), Toll-like receptor 2 (TLR2), and nuclear factor kappa B (NF-κB) in vitro. These results indicate that *L. crispatus* may play a role in modulating cervical inflammation in CI by suppressing the TLR/NF-κB signaling pathway, potentially contributing to a more stable cervical microenvironment during pregnancy.

**Supplementary Information:**

The online version contains supplementary material available at 10.1186/s12866-025-04203-0.

## Introduction

Cervical Incompetence (CI) involves cervical shortening and dilation during pregnancy [1], raising the risk of perinatal complications and threatening maternal and infant health [2]. Dysbiosis of the cervicovaginal microbiota may contribute to negative pregnancy outcomes in CI cases [3–5]. CI affects about 0.5–1% of pregnancies, increasing to 8% in women with recurrent miscarriages [6, 7].

Patients with CI frequently do not exhibit anatomical abnormalities of the cervix, and the prevailing treatment strategies are both pharmacological and surgical. Cervical cerclage, which can be executed via transvaginal or transabdominal approaches, is regarded as the sole efficacious surgical intervention for CI during pregnancy [8, 9]. This procedure substantially extends gestational duration and enhances neonatal survival rates by placing a ligature around the cervix to mechanically prevent premature dilation [8, 10, 11]. In a typical pregnancy, the cervix acts as a competent barrier, maintaining intrauterine sterility; cervical cerclage augments this function by providing mechanical defense against ascending infections and the resultant inflammation [12].

Cervical canal relaxation and the reduction of mucus plugs are indicative of functional abnormalities in patients with CI [12, 13]. Notably, 80% of acute CI cases are closely linked to infections of the amniotic cavity, which trigger inflammatory responses in cervical tissue. This inflammation leads to collagen degradation and remodeling of the extracellular matrix, thereby aggravating the pathological progression of CI [14]. Cervical mucus serves as a natural barrier and is vital for the normal functioning of the cervix, with its microbial equilibrium being crucial for maintaining cervical health [15]. CI is strongly associated with dysbiosis of cervical mucus, in stark contrast to the *Lactobacillus*-dominated microbiota and the acidic, antimicrobial environment characteristic of normal cervical physiology, which inhibits the proliferation of pathogenic bacteria [16]. Zhou et al. indicates that, compared to pre-cerclage microbiota, the post-cerclage vaginal microbiota showed greater diversity, increased *Lactobacillus*, and decreased Gardnerella, with improvements observed in both pregnancy outcomes and the vaginal environment [17]. Nevertheless, the effect of cervical cerclage on the microbiota within cervical mucus in patients with CI remains unclear. Furthermore, the precise mechanisms connecting the cervical mucus microbiota to CI require further exploration.

## Materials and methods

### Samples

The pregnant woman with CI was diagnosed by the Department of Obstetrics and Gynecology, the Shijiazhuang Fourth Hospital, and Cervical cerclage was performed. Pre-operative (PreOp) and Post-operative (PostOp) cervical mucus specimens were collected with patient’s informed consent. Ethical clearance was obtained from the Ethical Review Board of the Shijiazhuang Fourth Hospital (No. 2023028).

### Construction of16S rRNA amplicon sequencing library

The microbiotas of PreOp and PostOp cervical mucus were analyzed using high-throughput 16S rRNA gene sequencing. In brief, the 16S rRNA gene sequencing were performed using 16S rRNA gene PCR primers (Forward Primer 5‘-TCG TCG GCA GCG TCA GAT GTG TAT AAG AGA CAG CCT ACG GGN GGC WGC AG-3’, Reverse Primer 5‘-GTC TCG TGG GCT CGG AGA TGT GTA TAA GAG ACA GGA CTA CHV GGG TAT CTA ATC C-3’). The DNA extracted from cervical mucus by Trizol, and high-throughput sequencing was performed on the library using Illumina and other platforms to obtain a large number of 16 S rRNA gene sequence data. The raw sequencing data have been uploaded to the NCBI Sequence Read Archive (SRA) with the Accession Number PRJNA1222091.

Quantitative Insights for Microbial Ecology (QIIME) (v1.91) was used for sequence splicing and taxonomic analysis, and Uparse and RDP Classifiers were used for OTU clustering and sequence classification annotation. Raw FASTQ files were de-multiplexed with a custom perl script, quality-filtered using fastp v0.19.6, and merged with FLASH v1.2.11. The criteria were: (i) reads were truncated at an average quality score < 20 over a 50 bp window, discarding those shorter than 50 bp or with ambiguous characters; (ii) only sequences with overlaps > 10 bp and a maximum mismatch ratio of 0.2 were assembled; (iii) samples were identified by barcode and primers, with exact barcode matching and up to 2 mismatches in primer matching. Then the optimized sequences were clustered into OTUs using Usearch 11 with 97% sequence similarity level. The most abundant sequence for each OTU was selected as a representative sequence. To minimize the effects of sequencing depth on alpha and beta diversity measure, the number of 16 S rRNA gene sequences from each sample were rarefied to 59,433.

### Bacterial culture and supernatant separation

The *Lactobacillus crispatus* (*L. crispatus*) (ATCC 33197) and Group B Streptococcus (GBS) (ATCC 13813), were obtained from Shanghai Luwei Technology company (Shanghai, China). They were cultured at 37 °C until colonies formed, then grown in liquid medium for 72 and 48 h, respectively. Centrifuge at 10,000 rpm at 4 ° C for 10 min. Supernatants were stored at − 20 °C for later use, and filtered through a 0.22 μm filter. In the antibiotic-free HcerEpic cell medium, dilute the bacterion-free supernatant to a gradient concentration of 1–30% v/v. The bacterial density was calculated using the fully automatic colony counter (Interscience, France), and the final concentration of the bacteria-free supernatant in each well (10^5^−10^7^ CFU/ml culture density).

### Cell culture

Human cervical epithelial cells (HcerEpic) were purchased from BeNa Culture Collection (Jiangsu, China), and were cultured in MEM medium with 10% fetal bovine serum (Invitrogen, USA), and 1% penicillin/streptomycin (Sigma-Aldrich, USA) at 37 °C with 5% CO2. HcerEpic were passaged when the cell density reached 80%, and cells in the logarithmic growth phase were selected for experiments. HcerEpics were plated at 2.0 × 10^5^ cells/dish for 24 h in vitro using normal saline (negative control group), 25% *L. crispatus* supernatant, 25% GBS supernatant, and 25% *L. crispatus* supernatant + 25% GBS supernatant respectively. The cells collected from the three repeated experiments were centrifuged, and the cell culture supernatants were collected.

### CCK8

The CCK8 assay was conducted using the CCK-8 Cell Proliferation and Cytotoxicity Assay Kit (Solarbio, China). 5,000 cells were implanted in 100 µL culture medium and stimulated with bacterial supernatant of gradient concentration for 24 h. The 10 µL CCK8 detection solution was added to each well and incubated in CO_2_ incubator for 24 h. Three duplicate wells were set for each concentration, and the experiment was repeated three times. Absorbance value was detected at 450 nm to calculate cell proliferation ability.

### Quantitative reverse transcription polymerase chain reaction (qRT-PCR)

Total RNA was isolated from HcerEpic treated with bacterial solution supernatant using Trizol reagent (Thermo Fisher, USA) according to the manufacturers’ protocols. The RNA was reverse-transcribed into cDNA using PrimeScript cDNA Synthesis kit (Takara, Japan), according to the operator’s manual. Each target transcript was amplified using specific primers, as shown in supplemental Table S1. The qRT-PCR uses 2 µL cDNA as the template and the final reaction volume is 20 µL. Each reaction consisted of a PCR reaction that was pre-denatured at 95 for 10 min, pre-denatured at 95 °C for 10 min, then denatured at 95 °C for 10 s, and annealed for 1 min at 60 °C for 40 cycles. The relative gene expression level was calculated using the 2^−∆∆^Ct method, with fold-change values provided in Supplementary Table 2. Gene expression levels were normalized against the expression level of GAPDH.

### Western blotting

The HcerEpic treated with bacterial supernatant were dissolved in ice-cold RIPA lysis buffer (Servicebio, China) containing a protease and a phosphatase inhibitor cocktail (Servicebio, China). Cell lysate samples of three repeated experiments were centrifuged at 12,000×g for 10 min at 4 °C and heated at 100 °C for 5 min, and the protein concentration of cell lysate was quantified by bicinchoninic acid (BCA) method, according to the manufacturers’ protocols of kits (Thermo, USA). Equivalent amounts of cell lysates were then exposed to 10% SDS-PAGE gels, followed by transfer onto nitrocellulose membranes (Bio-Rad, Hercules, CA, USA). The membranes were blocked with 5% skimmed milk in Tris-buffered saline with Tween-20 (TBST) for 1 h at room temperature and then probed overnight at 4 °C with the respective primary antibodies including anti-TLR4 (1:1000; BIOSS, China), anti-TLR2 (1:1000; BIOSS, China), anti-NF-κB (1:1000; BIOSS, China), anti-GAPDH (1:1000; Proteintech, china), followed by incubation with horseradish peroxidase-conjugated secondary antibodies (Proteintech, china). The protein signals were detected using an enhanced chemiluminescence system detection kit (servicebio, China).

### Enzyme-linked immunosorbent assay (ELISA)

The HcerEpic treated with bacterial supernatants for 24 h, and collected the cell supernatants from three repeated experiments. The cell supernatant levels of IL-1β, IL-6 and TNF-α were analyzed using a commercial ELISA kit (R&D Systems, Minneapolis, MN, USA) according to the manufacturer’s instructions. The absorbance value of the cell supernatant is read at 450 nm wavelength.

### Statistical analysis

The microbial diversity and Venn analysis diagrams were performed by using the R packages, and the statistical analyses and plots were performed by GraphPad Prism v7.0. Statistical differences between experimental groups were determined using one-way ANOVA, the Mann-Whitney U test was used to compare the differences between the two groups.

## Results

### The abundance and structure of microbial communities between preop and PostOp cervical mucus samples

**Fig. 1:**
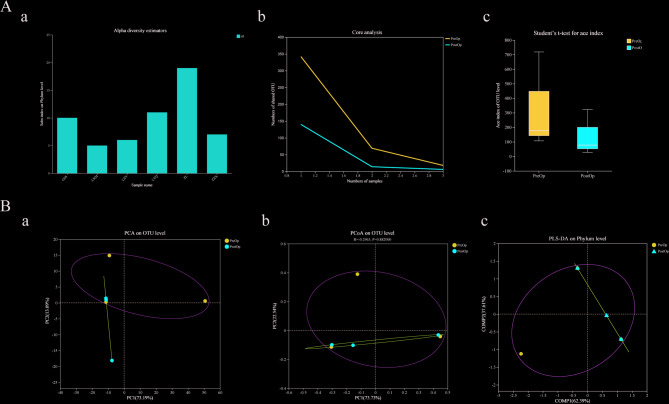
Characteristics of cervical mucus microbial community in the PreOp and PostOp. (A) Alpha diversity analysis. Sample species richness sobs index (a), sample core species number analysis (b), Alpha diversity index inter-group difference test (c); (B) Beta diversity analysis. Principal Component Analysis (PCA) on OUT level(a), principal co-ordinates analysis (PCoA) on OUT level (b), Partial Least Squares Discriminant Analysis (PLS-DA) on family level (c)

We assessed the species abundance and community structure of cervical mucus samples using alpha diversity and beta diversity analyses, respectively. The sobs index at the phylum level for each sample indicated the abundance of microbial populations (Fig. 1A-a). As sample numbers increased, the number of core species in both PreOp and PostOp samples gradually decreased; however, the number of core species in PreOp samples remained higher than that in PostOp samples (Fig. 1A-b). The ace index of PreOp and PostOp groups was compared to reflect the effect of surgery on species richness and average diversity. The PreOp species richness was higher than PostOp species richness, though the difference in microbial community diversity between the samples was not statistically significant (*p* > 0.05) (Fig. 1A-c).

Principal component analysis (PCA) at the operational taxonomic unit (OTU) level was performed to visualize structural differences in microbial communities between PreOp and PostOp samples. The two principal components, PC1 and PC2, contributed 73.19% and 13.09% to the dataset variation, respectively (Fig. 1B-a). An unsupervised learning method, principal coordinates analysis (PCoA), was used to evaluate similarities and differences between microbial communities, with PC1 and PC2 contributing 62.39% and 37.61%, respectively. Microbial community structure changes before and after surgery were not statistically significant (Fig. 1B-b). Using the supervised learning method PLS-DA at the phylum level, we differentiated microbial communities in different groups. COMP1 and COMP2 contributed 73.73% and 22.54% to the maximum data variation, respectively (Fig. 1B-c).

### Microbial functional diversity and dysbiosis dynamics in preop and PostOp cervical mucus samples

**Fig. 2:**
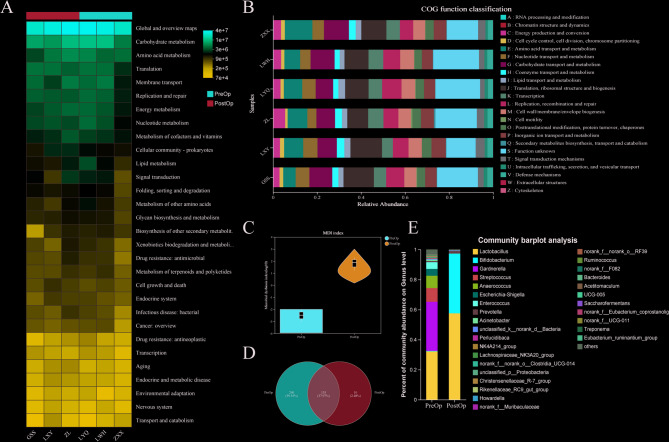
Analysis of microbial load, microbial diversity and functional enrichment of cervical mucus in the PreOp and PostOp. **A** KEGG pathway analysis of differential microbiota. **B** GO functional enrichment analysis of differential microbiota. **C** MDI index comparison. **D** Venn diagram of unique OTUs in PreOp and PostOp samples. **E** Microflora composition analysis

In the heatmap, red denotes high abundance, whereas blue signifies low abundance. The saturation of the color serves as a visual indicator of the relative abundance of diverse microbial functional pathways, spanning from fundamental metabolic processes, such as carbohydrate and amino acid transport and metabolism, to more intricate functions like translation, membrane transport, replication and repair, and signal transduction (Fig. 2A). Using COG functional classification, the gene-level functional diversity of microbial communities was emphasized, with distinct colors representing categories including defense mechanisms, chromosomal structure and dynamics, and energy production and conversion (Fig. 2B).

The microbial dysbiosis index (MDI) of PostOp cervical mucus samples was higher than that of PreOp samples, indicating an increased degree of dysbiosis (Fig. 2C). The Venn diagram shows that the PreOp samples contained 230 unique OTUs, suggesting the presence of numerous distinct microbial species before surgery. The PostOp samples contained 143 unique OTUs, revealing a significant shift in microbial community structure and the introduction of new microbial species (Fig. 2D). The dominant microflora in both PreOp and PostOp groups included *Lactobacillus*,* Bifidobacterium*,* Gardnerella*,* Streptococcus*,* and Anaerococcus*, with higher abundance of *Lactobacillus* and *Bifidobacterium* in the PostOp group (Fig. 2E).

### Effects of *L. crispatus* and GBS supernatants on HcerEpic cell morphological growth in vitro

**Fig. 3:**
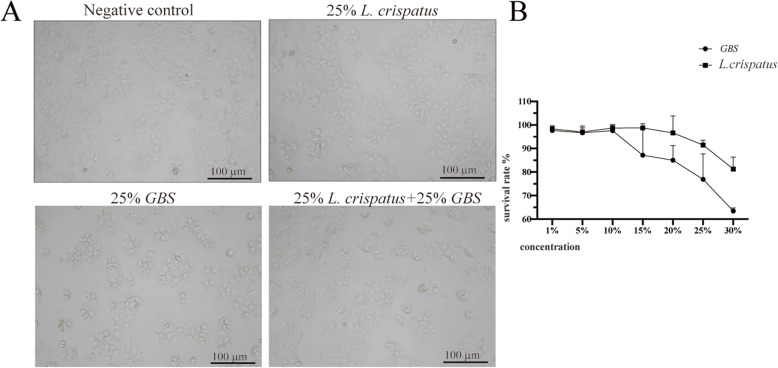
HcerEpic cells were stimulated with bacterial supernatants in vitro. **A** Microscopic images of cells after bacterial supernatants treatment. **B** Cell viability as measured by CCK8 assay for 24 h post-stimulation

To observe the effects of *L. crispatus* and GBS supernatants on the morphology and growth of cervical epithelial cells, we stimulated HcerEpic cells with varying concentrations of bacterial solutions. Under optical microscopy, cells in the negative control group exhibited regular morphology, clear boundaries, and an adherent, mostly polygonal shape. In the 25% GBS group, the cervical epithelial cells showed irregular shapes, uneven distribution, and areas of cell aggregation. In the group treated with 25% *L. crispatus* + 25% GBS supernatants, cell aggregation was reduced, mitigating the cytotoxic effect of 25% GBS on cervical epithelial cells (Fig. 3A). As the concentration of bacterial solutions increased, cell viability decreased, with GBS significantly reducing the viability of cervical epithelial cells (Fig. 3B).

### Expression levels of damage repair-related genes in HcerEpic cells stimulated by *L. crispatus* supernatants

**Fig. 4:**
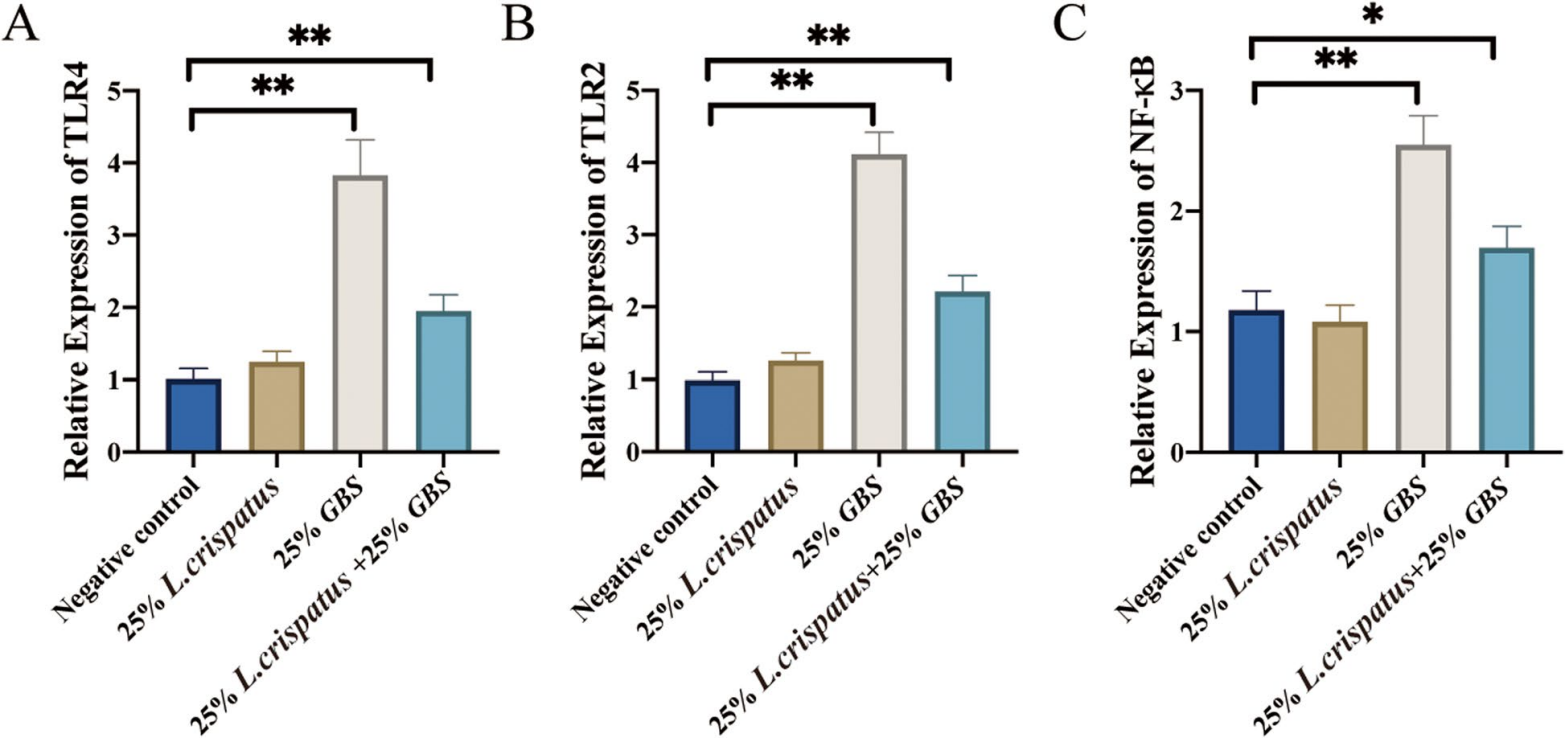
Relative gene expression levels of TLR4, TLR2, and NF-κB in HcerEpic stimulated with bacterial supernatants in vitro

To examine changes in damage repair-related gene expression after bacterial supernatants stimulation, we measured the expression levels of TLR4, TLR2, and NF-κB genes. Compared to the negative control, the 25% GBS treatment group showed a significant increase in TLR4 and TLR2 expression (*p* < 0.01) (Fig. 4A). However, the combined treatment of 25% GBS + 25% *L. crispatus* significantly reduced TLR4 and TLR2 expression levels (*p* < 0.01) (Fig. 4B). Additionally, the 25% GBS group significantly upregulated NF-κB expression (*p* < 0.01), whereas the 25% GBS + 25% *L. crispatus* treatment group significantly downregulated NF-κB expression (*p* < 0.05) (Fig. 4C).

### Expression levels of damage repair-related proteins in HcerEpic cells stimulated by *L. crispatus* supernatants

**Fig. 5:**
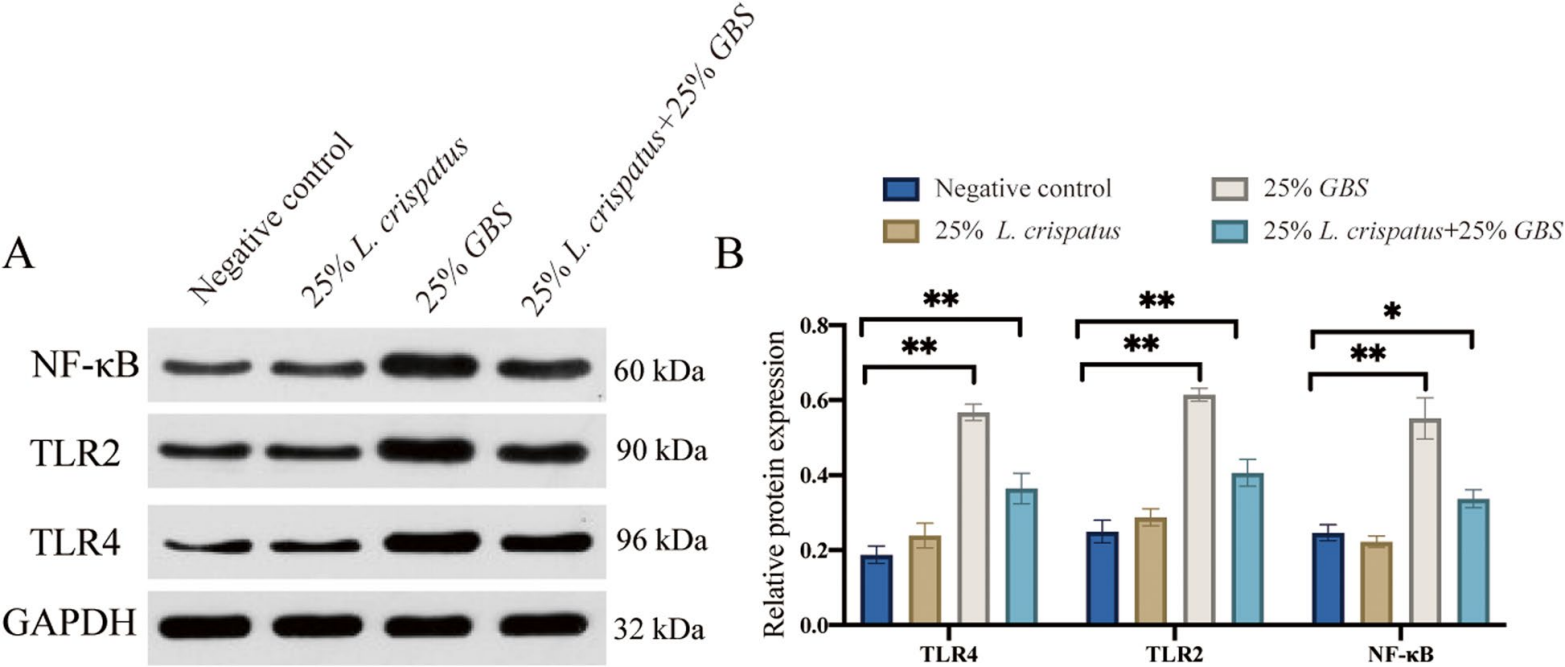
Relative expression of protein in c HcerEpic stimulated by bacterial supernatants in vitro. **A** Protein band of TLR4, TLR2, and NF-κB protein; **B** Statistical diagram of TLR4, TLR2, and NF-κB protein expression levels

Following the observed changes in gene expression of TLR4, TLR2, and NF-κB, we further examined their protein levels (Fig. 5A). Compared to the negative control, treatment with 25% *L. crispatus* did not significantly alter TLR4, TLR2, and NF-κB protein expression (*p* > 0.05), whereas 25% GBS significantly increased TLR4, TLR2, and NF-κB protein levels (*p* < 0.01). However, the combined treatment of 25% *L. crispatus* and 25% GBS significantly reduced TLR4 and TLR2 protein expression (*p* < 0.01) and lowered NF-κB protein levels (*p* < 0.05) (Fig. 5B).

### Cytokine levels in the supernatant of HcerEpic cells stimulated by *L. crispatus* supernatants

**Fig. 6:**
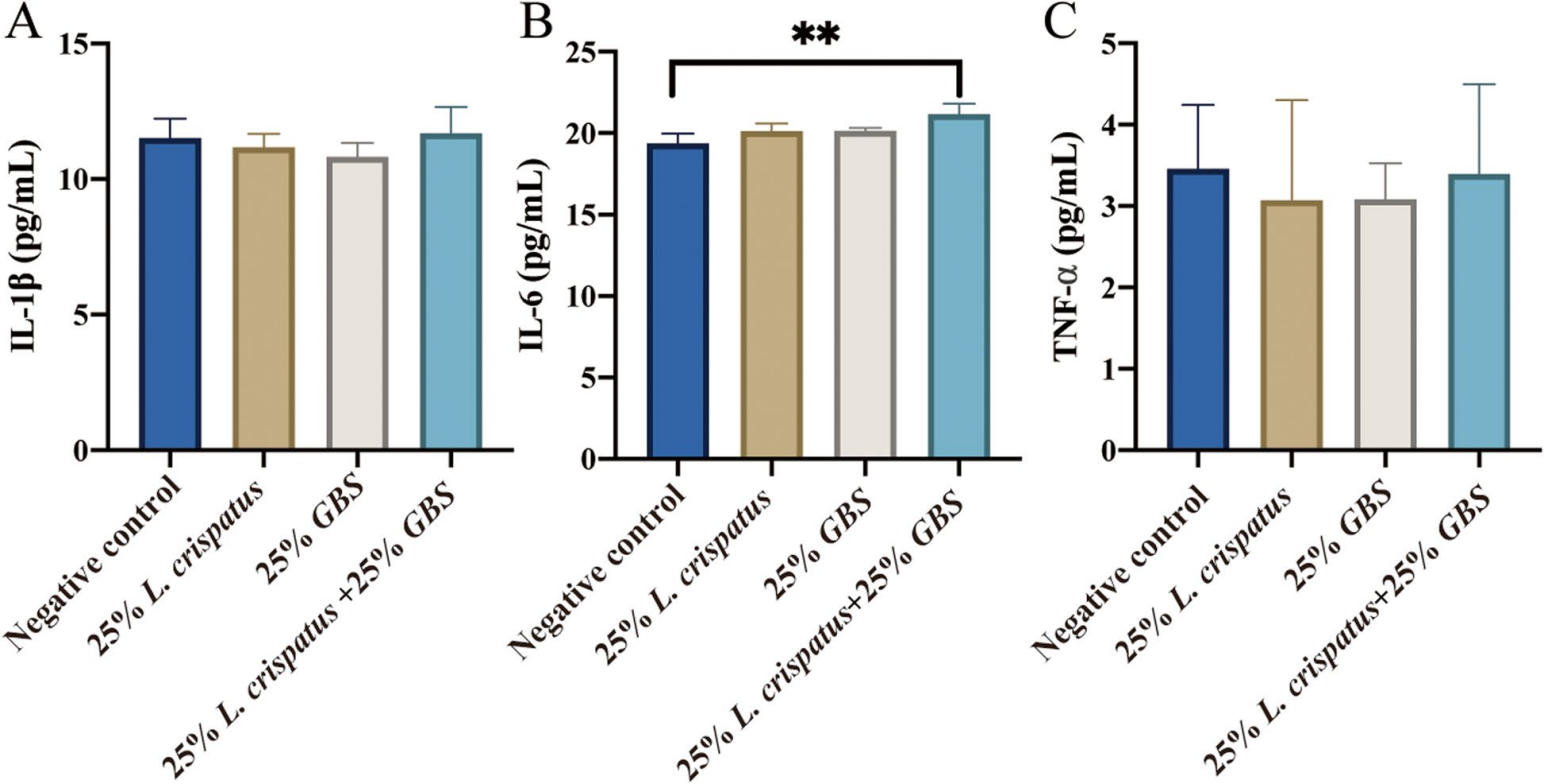
Changes in IL-1β, IL-6, and TNF-α cytokine levels in the supernatant of HcerEpic stimulated by bacterial supernatants for 24 h

To assess the inflammatory response in cervical epithelial cells following stimulation with *L. crispatus* and GBS supernatants, we measured IL-1β, IL-6, and TNF-α cytokine levels in the cell culture supernatants. There were no significant differences in IL-1β and TNF-α levels across treatment groups compared to the negative control (*p* > 0.05) (Fig. 6A, C). However, compared to the negative control, the combined stimulation of cervical epithelial cells with GBS and *L. crispatus* led to an increase in IL-6 levels in the supernatant (*p* < 0.01) (Fig. 6B). The increased IL-6 levels may represent a cellular response to microbial stimulation, aiming to enhance local immune defenses against potential infections or damage. This response aids in strengthening local immunity, eliminating potential pathogens, and promoting tissue repair and regeneration.

## Discussion

In both non-pregnant and pregnant states, the endocervical canal secretes substances like mucus and cytokines, crucial for structural support and immune protection during pregnancy [18, 19]. The imbalance of microorganisms in cervical mucus makes cervical tissue more susceptible to infection, inducing inflammatory responses in cervical tissue, leading to collagen degradation and extracellular matrix remodeling to aggravate CI [20]. This study found that cervical cerclage can change the microbiome of cervical mucus in CI patients. *L. crispatus* may modulate immunity via the TLR/NF-κB pathway in vitro. Thus, monitoring and managing reproductive tract microbiota is essential for preventing childbirth complications.

Increased microbial diversity indicates that reproductive tract ecological disorders are associated with poor reproductive outcomes, while *Lactobacillus spp.* is associated with reproductive health [21]. Women with cervical incompetence (CI) have reduced vaginal *Lactobacillus spp.* abundance and increased colonization with *Gardnerella spp.* and *Prevotella spp.*, and these microecological alterations are associated with worse cerclage outcomes, with higher bacterial densities in treatment failure cases than in full-term pregnancies [17, 22, 23]. Our study found that PreOp cervical mucus samples contained higher levels of Gardnerella than PostOp samples. The presence of Gardnerella may disrupt the normal microbiome balance within the reproductive tract, potentially triggering inflammation or infection and thus increasing the risk of preterm birth [24, 25]. Genital colonization by GBS in pregnant women is a major factor in adverse pregnancy outcomes such as preterm birth, premature rupture of membranes, chorionitis, and fetal infection [26, 27]. *Lactobacillus spp.* produces beneficial substances like lactic acid, which maintains the acidic environment of the reproductive tract, inhibiting the growth of harmful bacteria [28]. Additionally, *Lactobacillus spp.* induces autophagy in vaginal epithelial cells, killing intracellular microbes, reducing microbial diversity, and stabilizing the vaginal microbiome structure [29]. Consequently, preserving the predominance of *Lactobacillus spp.* within the reproductive tract microbiome is crucial for the prevention of preterm birth.

The female reproductive tract mucosa plays a key role in defending against microbial infections, which triggers immune responses by recognizing pathogenic molecular patterns such as Toll-like receptors (TLRs) [30]. TLRs, as key pattern recognition receptors, are vital in the immune system, recognizing microbial components such as lipopolysaccharides, urate crystals, and viral double-stranded RNA—collectively known as pathogen-associated molecular patterns (PAMPs) [31]. Activation of TLRs triggers a series of signaling pathways that activate NF-κB, which then translates to the nucleus, binds to specific DNA sequences, and induces the expression of inflammatory factors such as IL-1, IL-8, and TNF-α [32]. Selective activation of TLR 2 and TLR 4-dependent signaling pathways in mouse pregnancy tissues triggers preterm birth [33, 34]. In CI, excessive activation of the TLR/NF-κB pathway may play a significant role, as this pathway is also closely linked to physiological processes like proliferation and apoptosis in cervical cells [35].

Inflammatory cytokines play a major role in cervical remodeling and labor [36, 37]. Cervical tissues in CI patients exhibit higher expression levels of inflammatory factors, including IL-6, IL-8, and TNF-α, compared to those in normal pregnancies, which induce inflammatory responses in cervical tissues, leading to remodeling and structural changes that compromise cervical stability [20]. Elevated levels of cytokines such as IL-1β and IL-12 have been associated with increased TLR signaling [38, 39], which can lead to a robust inflammatory response to promote cervical ripening [40]. However, no effect on IL-1β and TNF-α levels in supernatant was observed. The different immune responses of cervical epithelial cells to microbial stimulation [41], and increasing the detection of related pro-inflammatory and anti-inflammatory factors is helpful for understanding the complexity of the reproductive tract immune environment [21].

This study has the following limitations: the sample size utilized in this study for characterizing the microbial flora is relatively limited, and could not cover the heterogeneity of pregnant women. In the future, the cohort needs to be expanded and confounding factors matched to generalize the conclusion to a larger population. In addition, animal models should be established to further verify the therapeutic potential of *Lactobacillus spp.* in future studies.

## Conclusion

This study indicates the critical role of the microbiota in cervical mucus in the pathogenesis of CI, specifically the balance between *Lactobacillus spp.* and pathogenic bacteria. We found significant changes in the cervical mucus microbiome before and after cervical cerclage, and *L. crispatus* may exhibit potential immunomodulatory effects through the TLR/NF-κB pathway. Combined stimulation of cervical epithelial cells with *L. crispatus* and GBS supernatants showed a reduction in inflammatory response, highlighting the potential therapeutic role of *L. crispatus* in restoring cervical immune homeostasis. Future in vivo studies should be conducted to further elucidate the mechanisms by which *L. crispatus* and other beneficial bacteria regulate reproductive tract immunity, with the ultimate goal of developing *L. crispatus* based interventions to protect patients with CI from both dysfunctional childbirth and premature birth.

## Supplementary Information

Supplementary Material 1. Supplementary Fig. 1 Full-length gel/blotting image of NF-kB protein. Supplementary Fig. 2 Full-length gel/blotting image of TLR4 protein. Supplementary Fig. 3 Full-length gel/blotting image of TLR2 protein. Supplementary table 1 Gene primer information. Supplementary table 2 The fold-change values of gene.

## Data Availability

The sample information and data involved in this study can be obtained by contacting the corresponding author with consent. The datasets have been uploaded to the NCBI Sequence Read Archive (SRA) with the Accession Number PRJNA1222091.
