# Supplementary material for: The role of cervical microbiome in cervical incompetence: insights from 16 S rRNA metagenomic sequencing
Source: BMC Microbiol. 2025 Aug 6;25:486. doi: 10.1186/s12866-025-04203-0 (PMC12326704; doi:10.1186/s12866-025-04203-0)
Supplement: Supplementary file 1 — Supplementary Material 1. Supplementary Fig. 1 Full-length gel/blotting image of NF-kB protein. Supplementary Fig. 2 Full-length gel/blotting image of TLR4 protein. Supplementary Fig. 3 Full-length gel/blotting image of TLR2 protein. Supplementary table 1 Gene primer information. Supplementary table 2 The fold-change values of gene. [file 12866_2025_4203_MOESM1_ESM.zip › Supplementary/Supplementary table.docx]

**Supplementary table**

Supplementary table 1Gene primer information

| Name | Primer |
| --- | --- |
| Homo GAPDH | Forward 5‘-TCAAGAAGGTGGTGAAGCAGG-3’ |
|  | Reverse 5‘-TCAAAGGTGGAGGAGTGGGT-3’ |
| Homo TLR4 | Forward 5‘-CCTGTCCCTGAACCCTATGA-3’ |
|  | Reverse 5‘-CCAGAACCAAACGATGGACT-3‘ |
| Homo TLR2 | Forward 5’-TGTGAAGAGTGAGTGGTGCA-3‘ |
|  | Reverse 5’-TACCCAAAATCCTTCCCGCT-3‘ |
| Homo NF-κB | Forward 5’-GGAGACATGAAACAGCTGGC-3‘ |
|  | Reverse 5’-TCGGTGTAGCCCATTTGTCT-3‘ |

Supplementary table 2 The fold-change values of gene

| Gene | Negative control | | | 25% Lactobacillus | | | 25% GBS | | | 25% Lactobacillus +25% GBS | | |
| --- | --- | --- | --- | --- | --- | --- | --- | --- | --- | --- | --- | --- |
|  | 1 | 2 | 3 | 1 | 2 | 3 | 1 | 2 | 3 | 1 | 2 | 3 |
| Homo GAPDH | 15.641 | 15.273 | 15.827 | 16.179 | 15.441 | 16.101 | 16.196 | 16.296 | 15.389 | 16.660 | 16.932 | 15.542 |
|  | 15.598 | 15.194 | 15.759 | 16.408 | 15.329 | 15.883 | 16.107 | 16.340 | 15.298 | 16.476 | 16.858 | 15.562 |
|  | 15.735 | 15.460 | 15.822 | 16.375 | 15.379 | 15.911 | 15.902 | 16.309 | 15.317 | 16.538 | 16.752 | 15.776 |
| Homo TLR4 | 28.413 | 27.918 | 28.603 | 29.103 | 28.240 | 28.173 | 27.296 | 27.418 | 26.228 | 28.392 | 28.986 | 27.528 |
|  | 28.622 | 28.029 | 29.064 | 28.873 | 27.892 | 28.480 | 27.151 | 27.222 | 25.977 | 28.285 | 29.160 | 27.440 |
|  | 28.484 | 27.901 | 28.916 | 28.795 | 28.104 | 28.338 | 27.056 | 27.171 | 26.011 | 28.303 | 28.658 | 27.413 |
| Homo TLR2 | 27.306 | 27.075 | 27.245 | 27.779 | 26.876 | 27.291 | 25.778 | 25.991 | 25.084 | 27.149 | 27.723 | 26.154 |
|  | 27.370 | 27.402 | 27.322 | 27.865 | 27.077 | 27.215 | 25.619 | 25.836 | 25.150 | 27.002 | 27.422 | 26.275 |
|  | 27.511 | 27.259 | 27.672 | 27.592 | 26.793 | 27.250 | 25.956 | 25.914 | 25.195 | 26.841 | 27.450 | 26.467 |
| Homo NF-κB | 24.305 | 23.388 | 24.219 | 24.952 | 23.796 | 24.163 | 23.542 | 23.299 | 22.707 | 24.334 | 24.984 | 23.862 |
|  | 24.130 | 23.613 | 24.011 | 24.861 | 24.108 | 24.315 | 23.216 | 23.383 | 22.557 | 24.282 | 24.551 | 23.458 |
|  | 24.442 | 23.753 | 24.134 | 25.082 | 24.155 | 24.437 | 23.589 | 23.685 | 22.822 | 24.330 | 24.507 | 23.745 |
