# Supplementary figures and images for: The role of cervical microbiome in cervical incompetence: insights from 16 S rRNA metagenomic sequencing
Source: BMC Microbiol. 2025 Aug 6;25:486. doi: 10.1186/s12866-025-04203-0 (PMC12326704; doi:10.1186/s12866-025-04203-0)

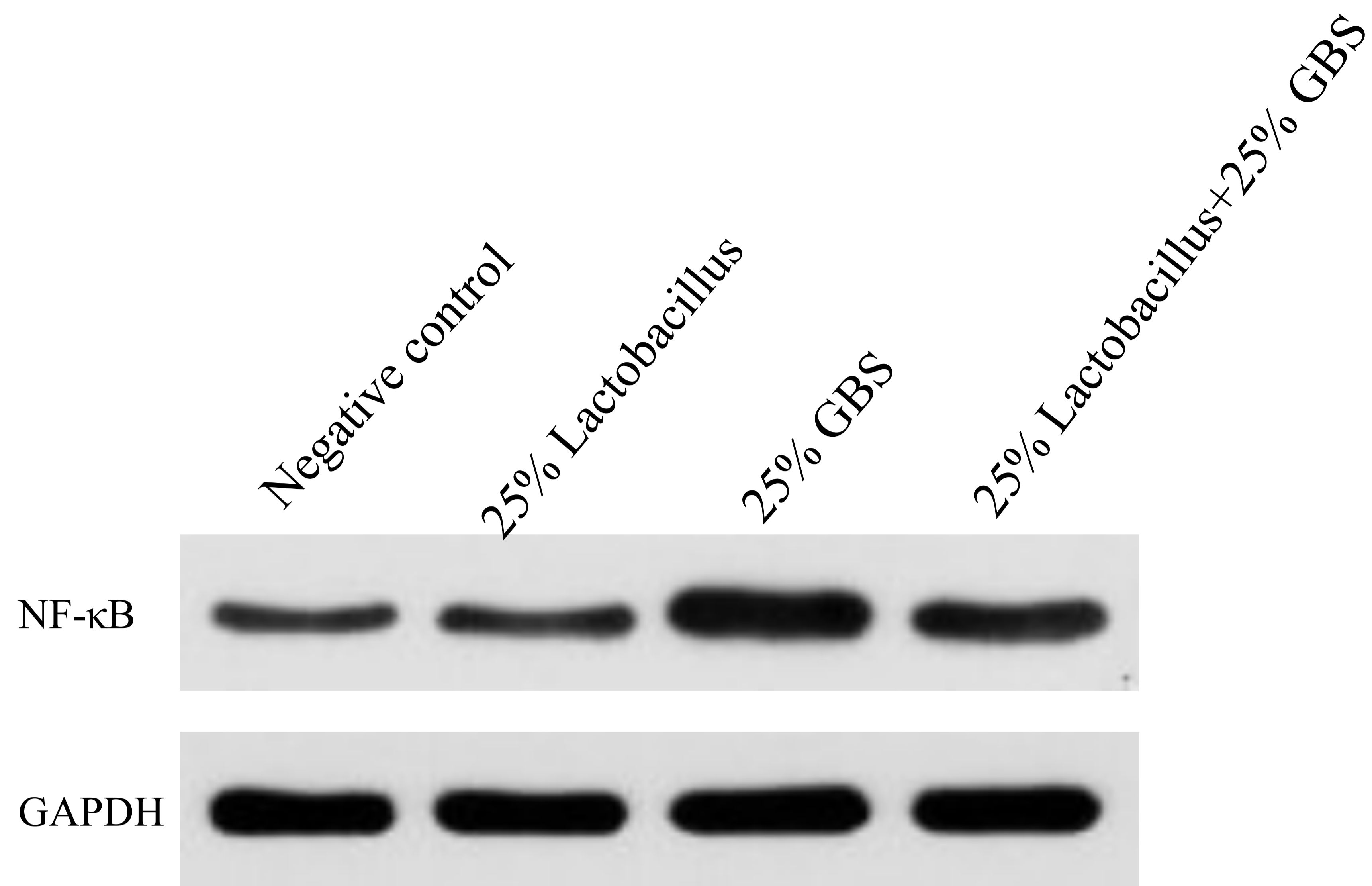

Complete gel image of GAPDH protein

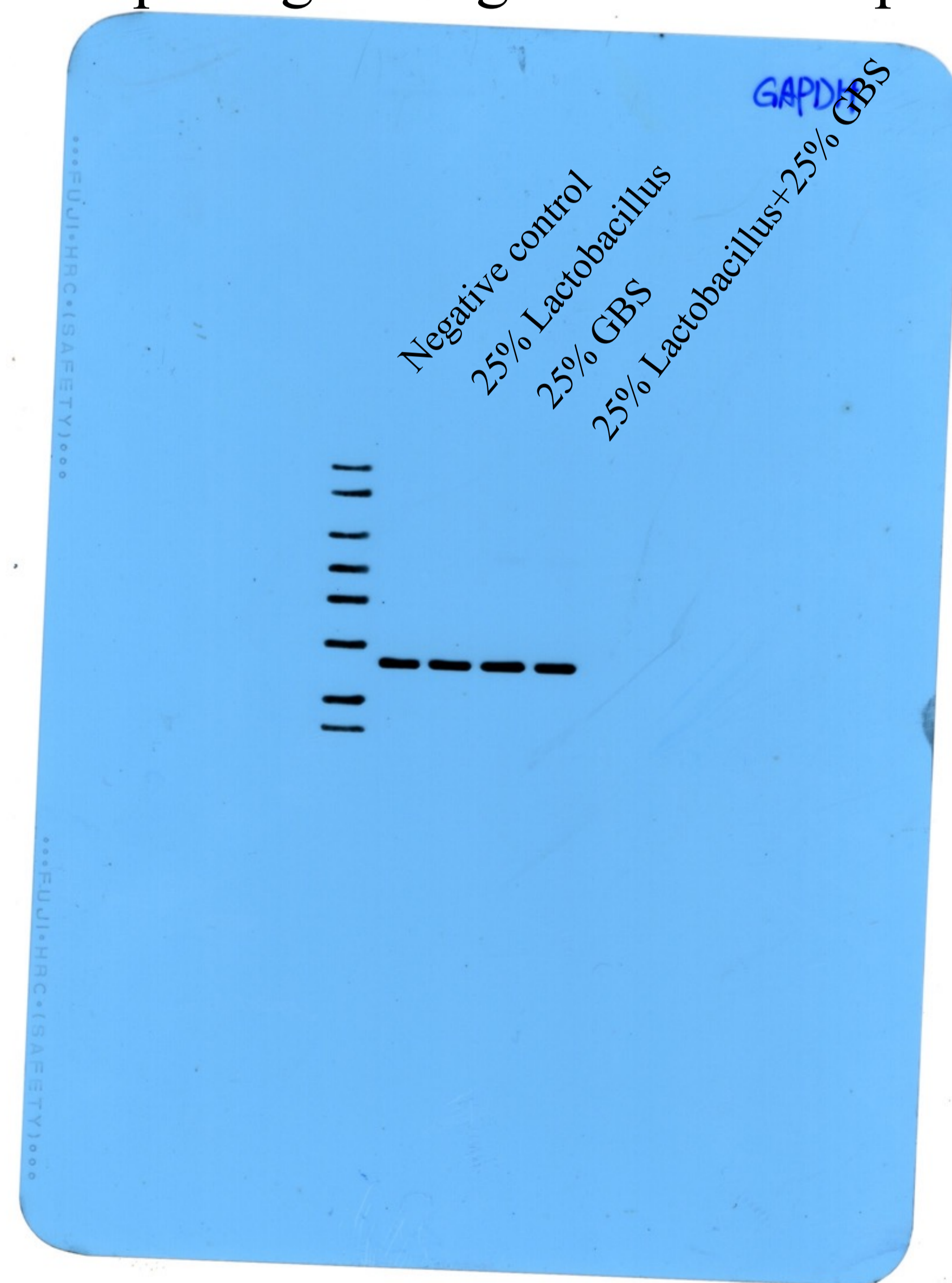

Complete gel image of NF- $\kappa$ B protein

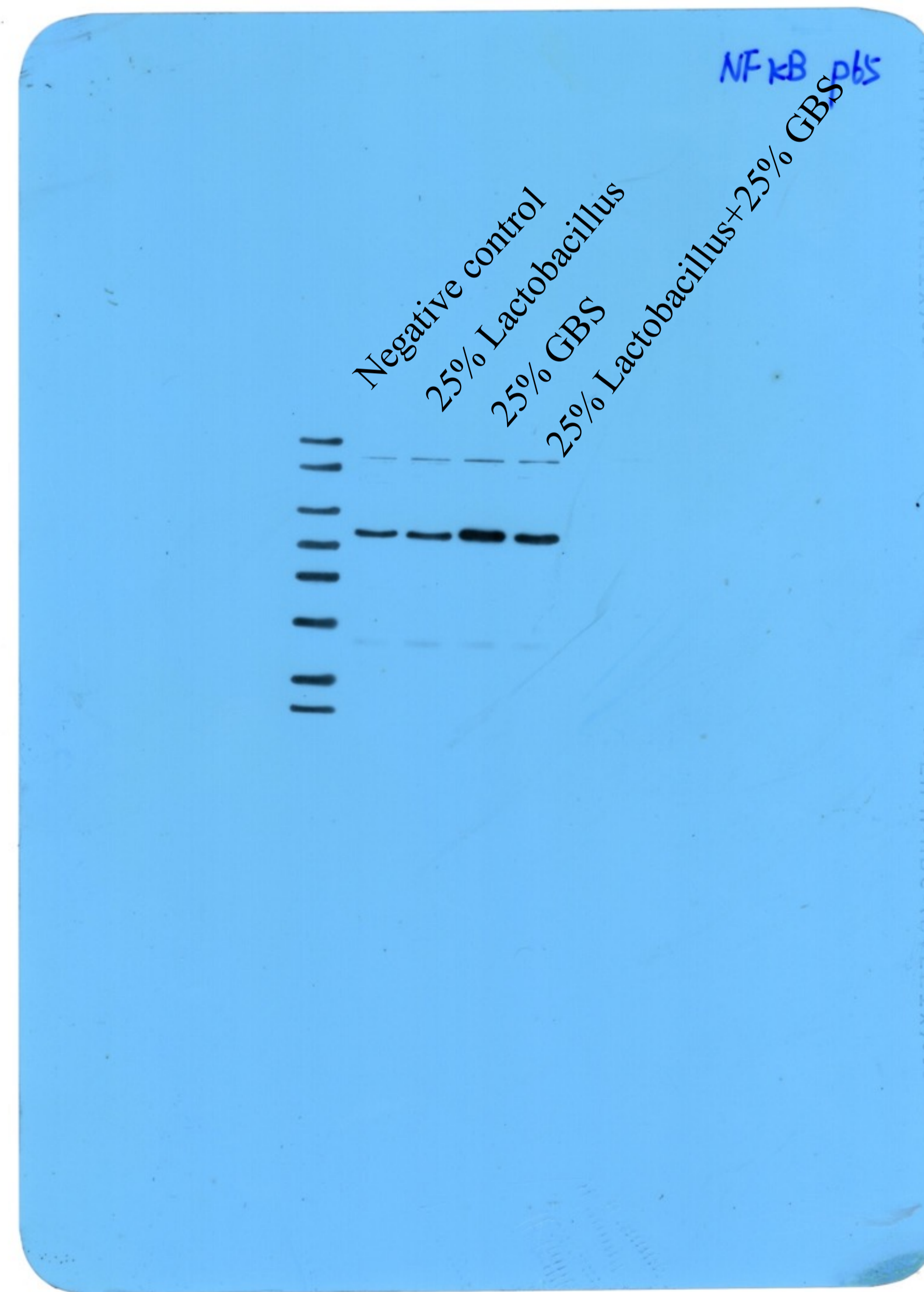

Merge

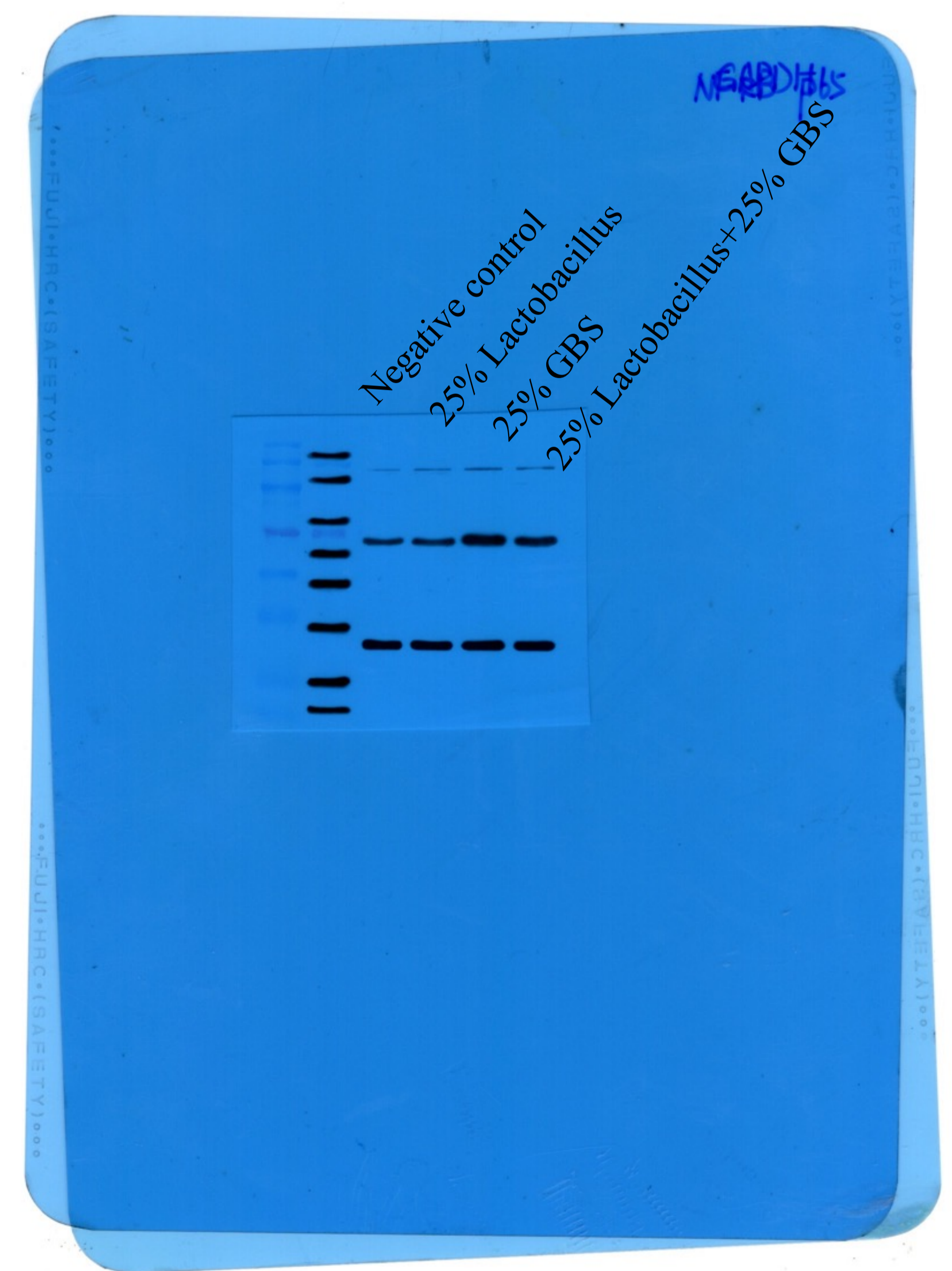

Supplement: Supplementary file 1 — Supplementary Material 1. Supplementary Fig. 1 Full-length gel/blotting image of NF-kB protein. Supplementary Fig. 2 Full-length gel/blotting image of TLR4 protein. Supplementary Fig. 3 Full-length gel/blotting image of TLR2 protein. Supplementary table 1 Gene primer information. Supplementary table 2 The fold-change values of gene. [file 12866_2025_4203_MOESM1_ESM.zip › Supplementary/Supplementary gel image NF-KB.pdf]

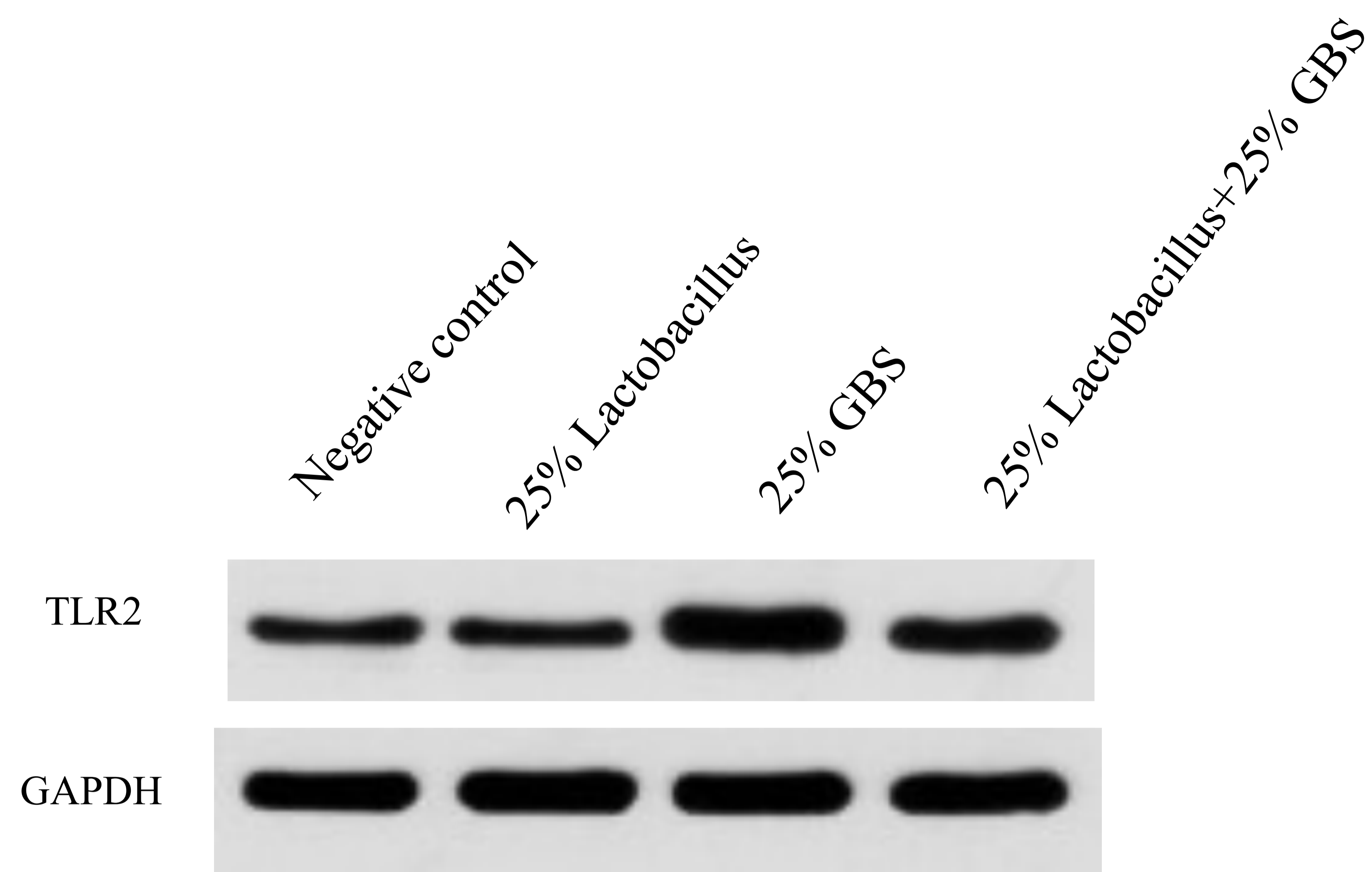

Complete gel image of GAPDH protein

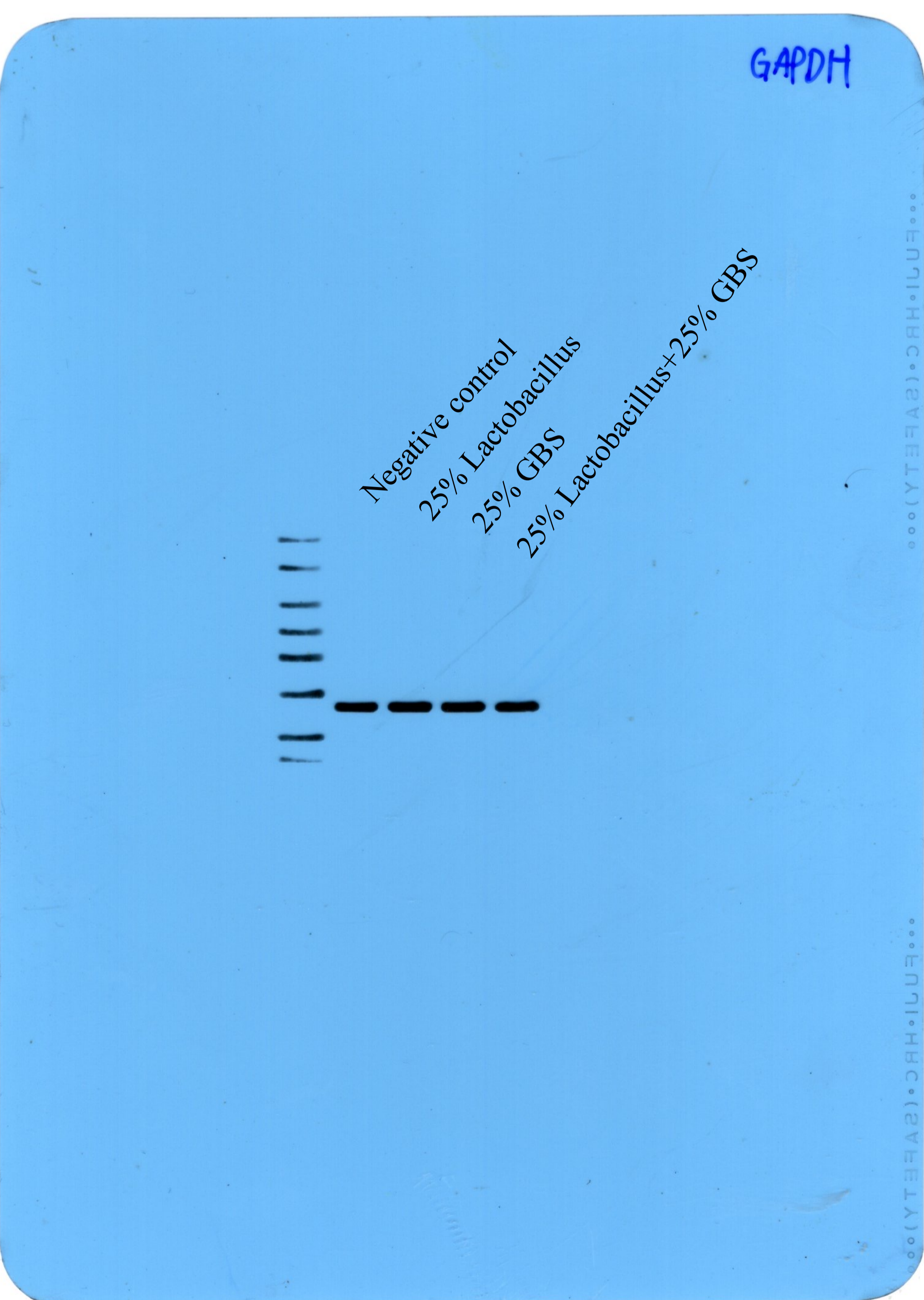

Complete gel image of TLR2 protein

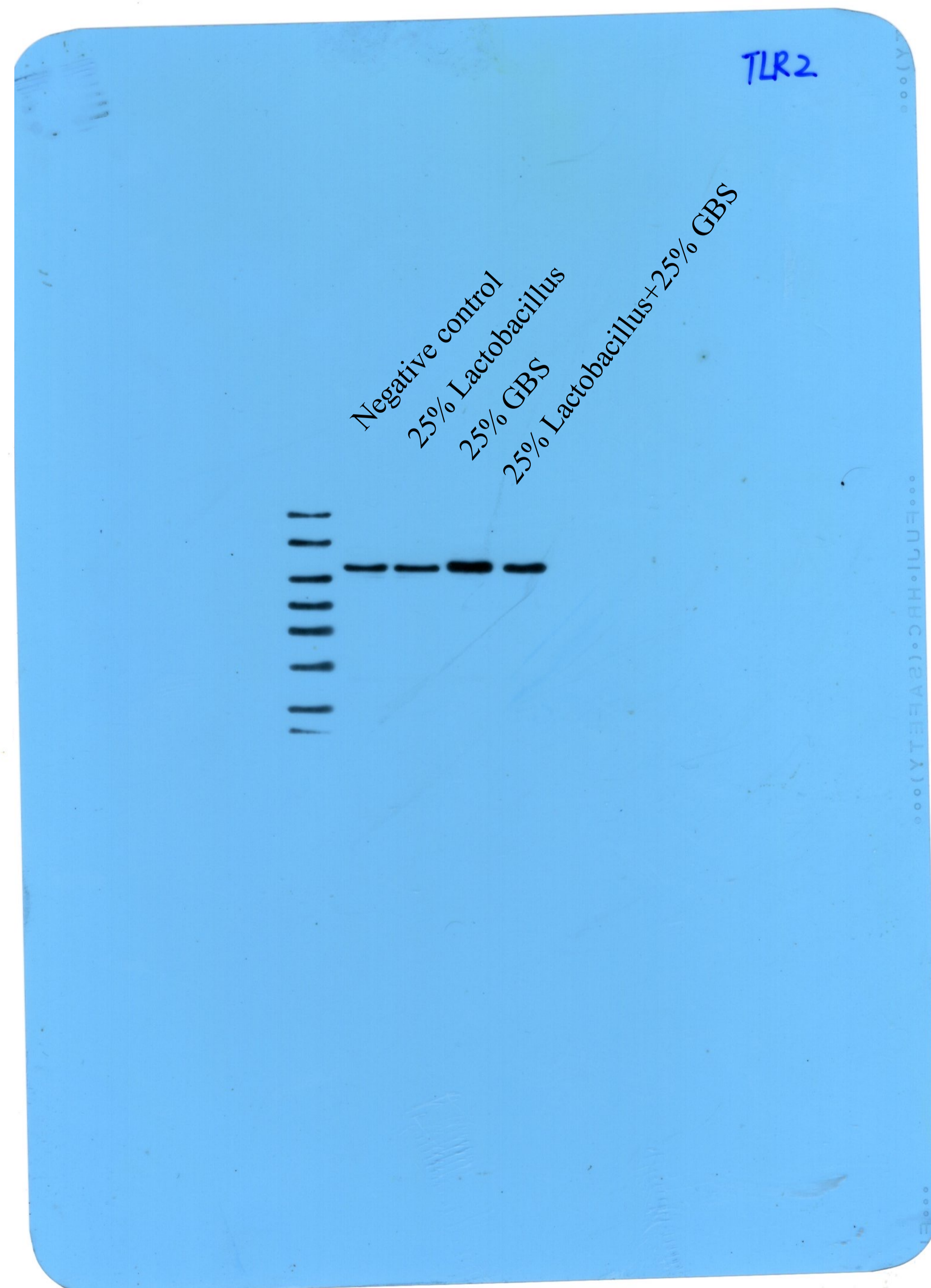

Merge

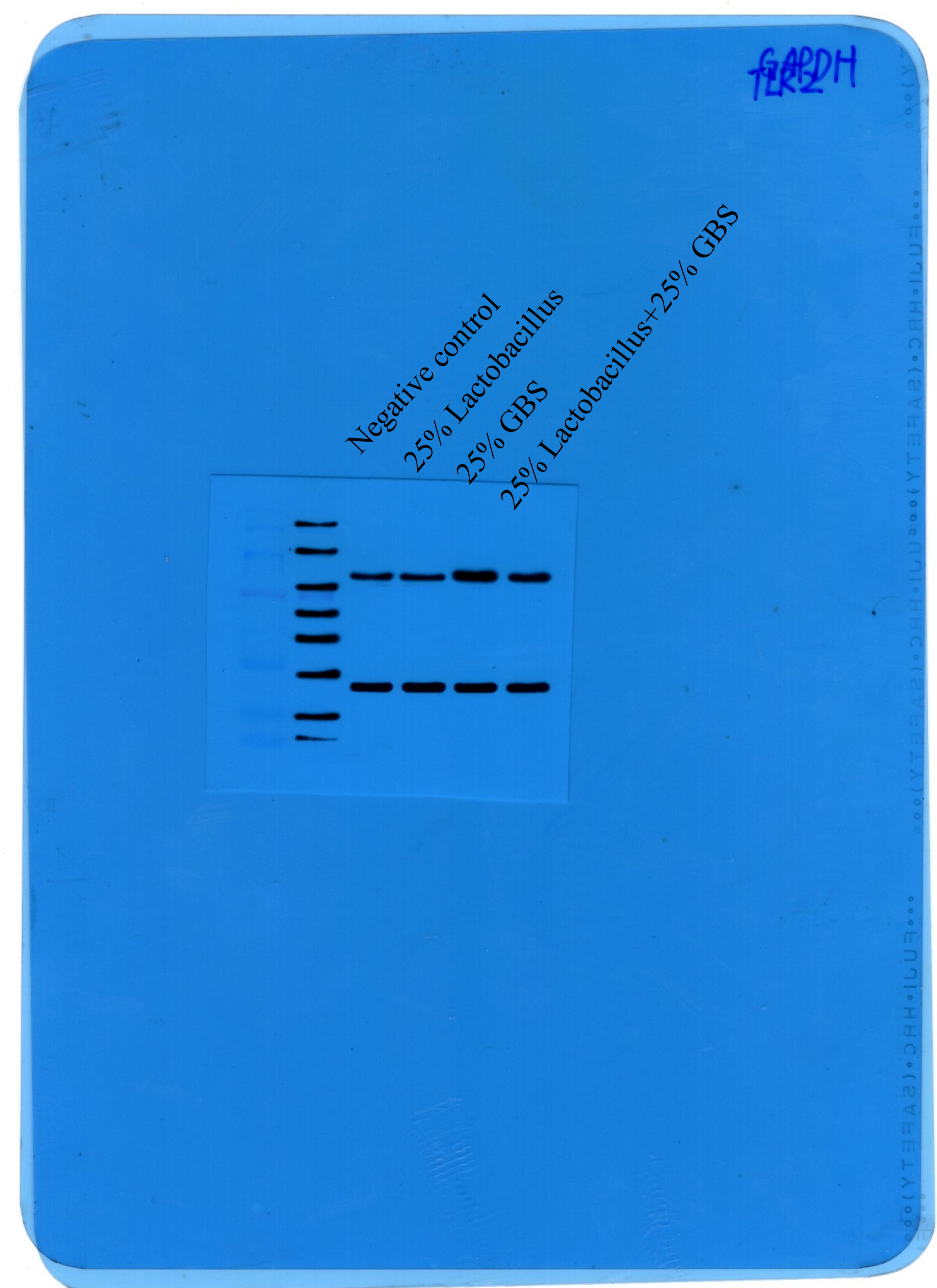

Supplement: Supplementary file 1 — Supplementary Material 1. Supplementary Fig. 1 Full-length gel/blotting image of NF-kB protein. Supplementary Fig. 2 Full-length gel/blotting image of TLR4 protein. Supplementary Fig. 3 Full-length gel/blotting image of TLR2 protein. Supplementary table 1 Gene primer information. Supplementary table 2 The fold-change values of gene. [file 12866_2025_4203_MOESM1_ESM.zip › Supplementary/Supplementary gel image TLR2.pdf]

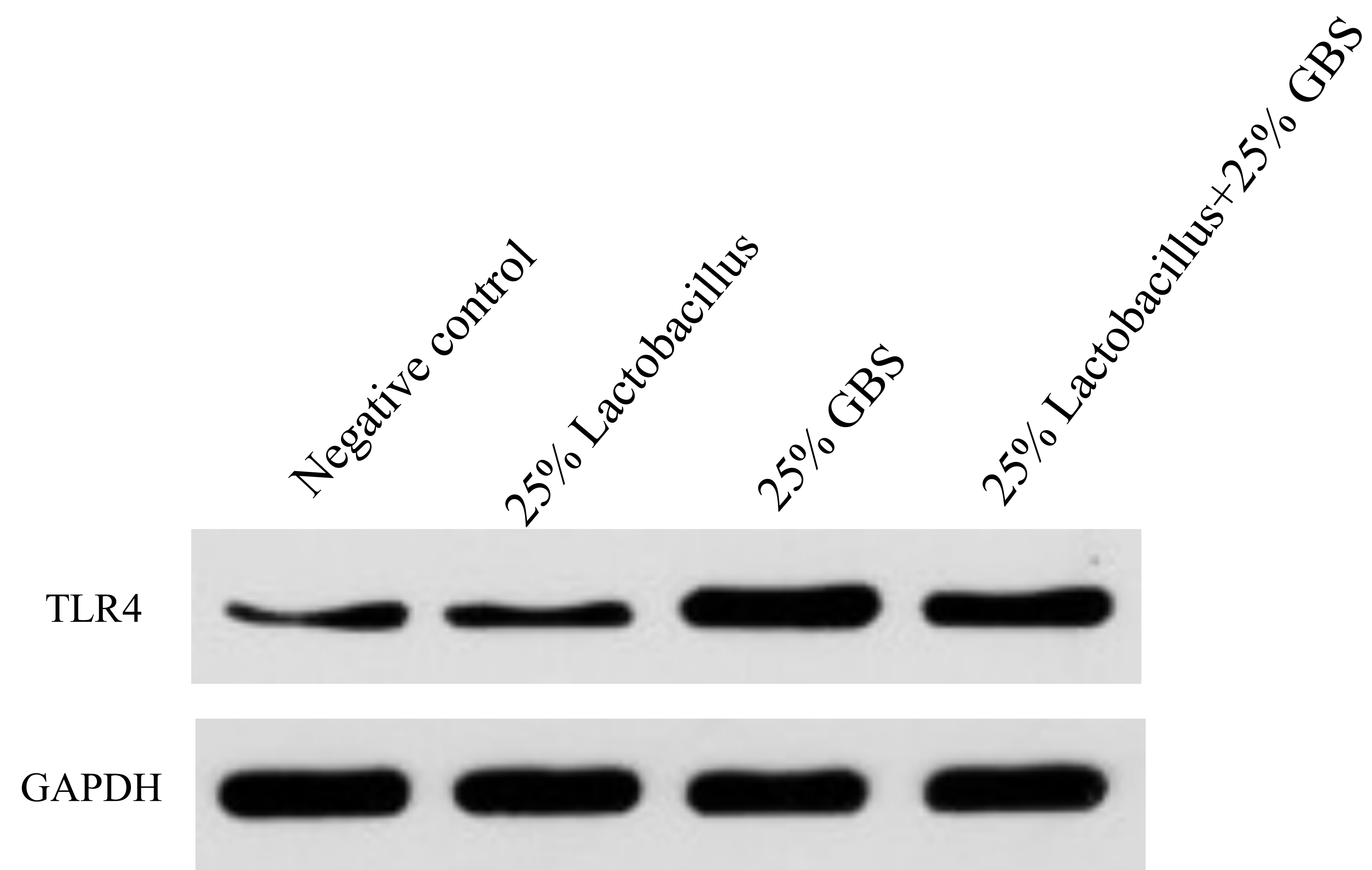

Complete gel image of GAPDH protein

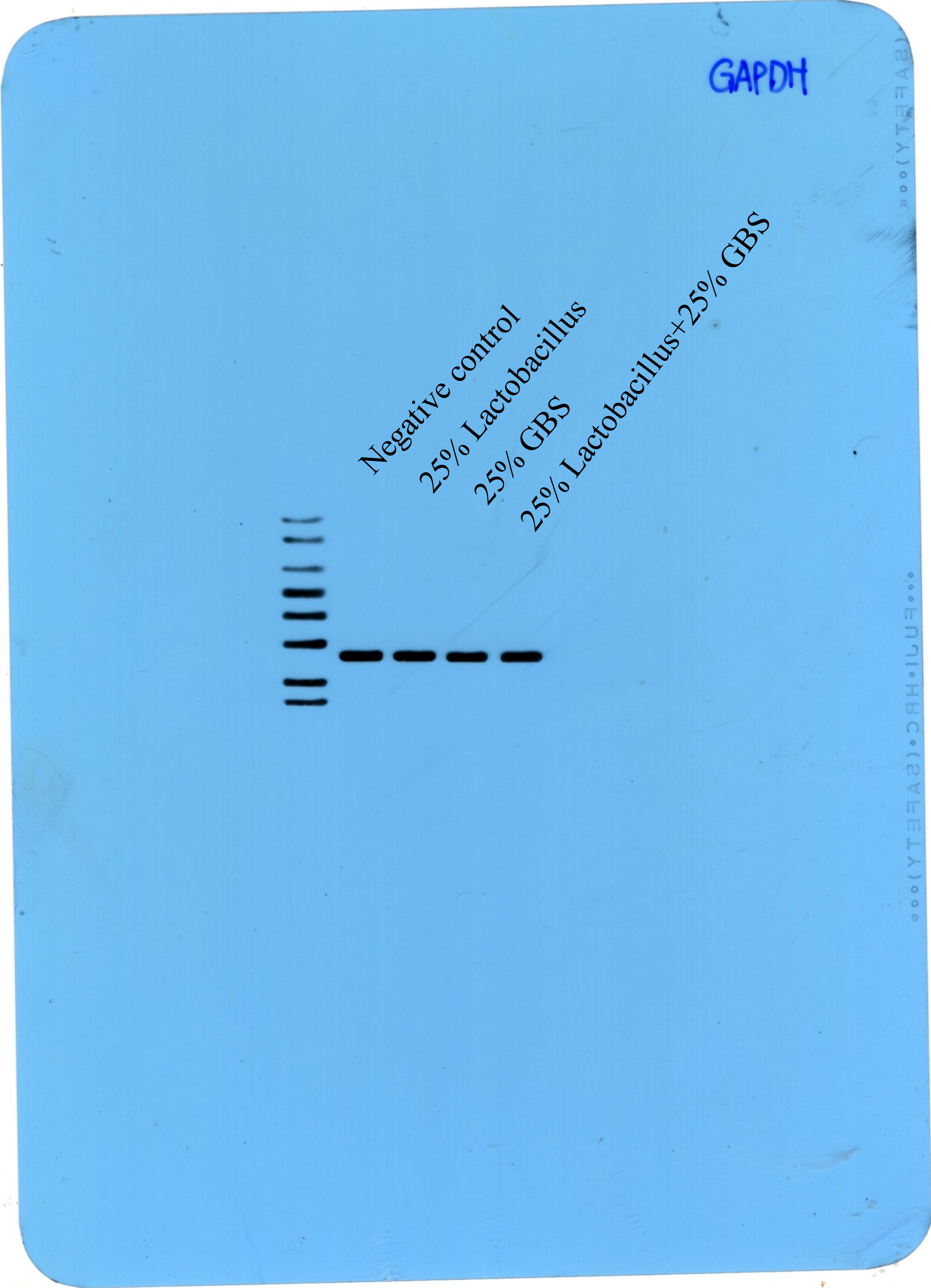

Complete gel image of TLR4 protein

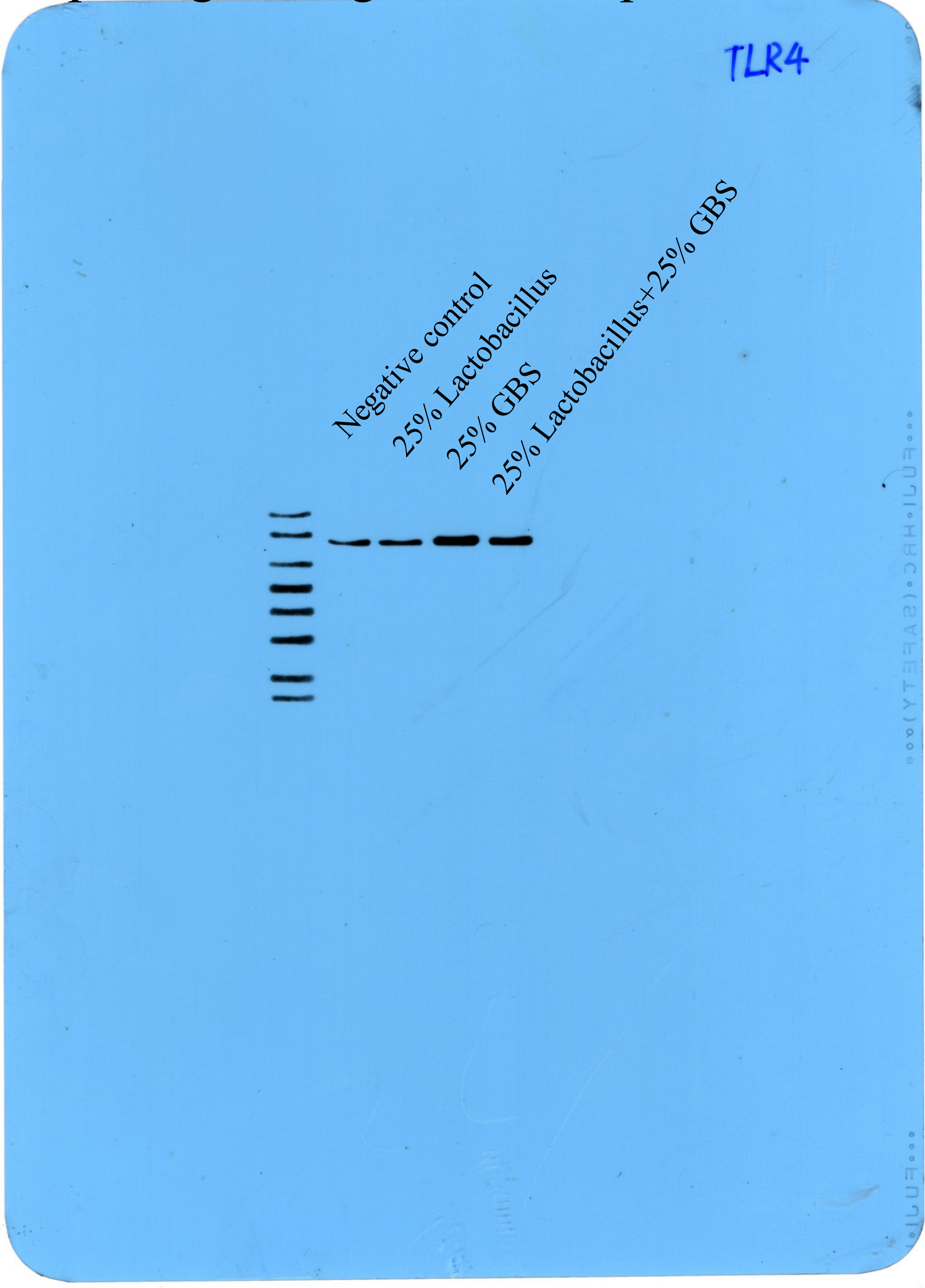

Merge

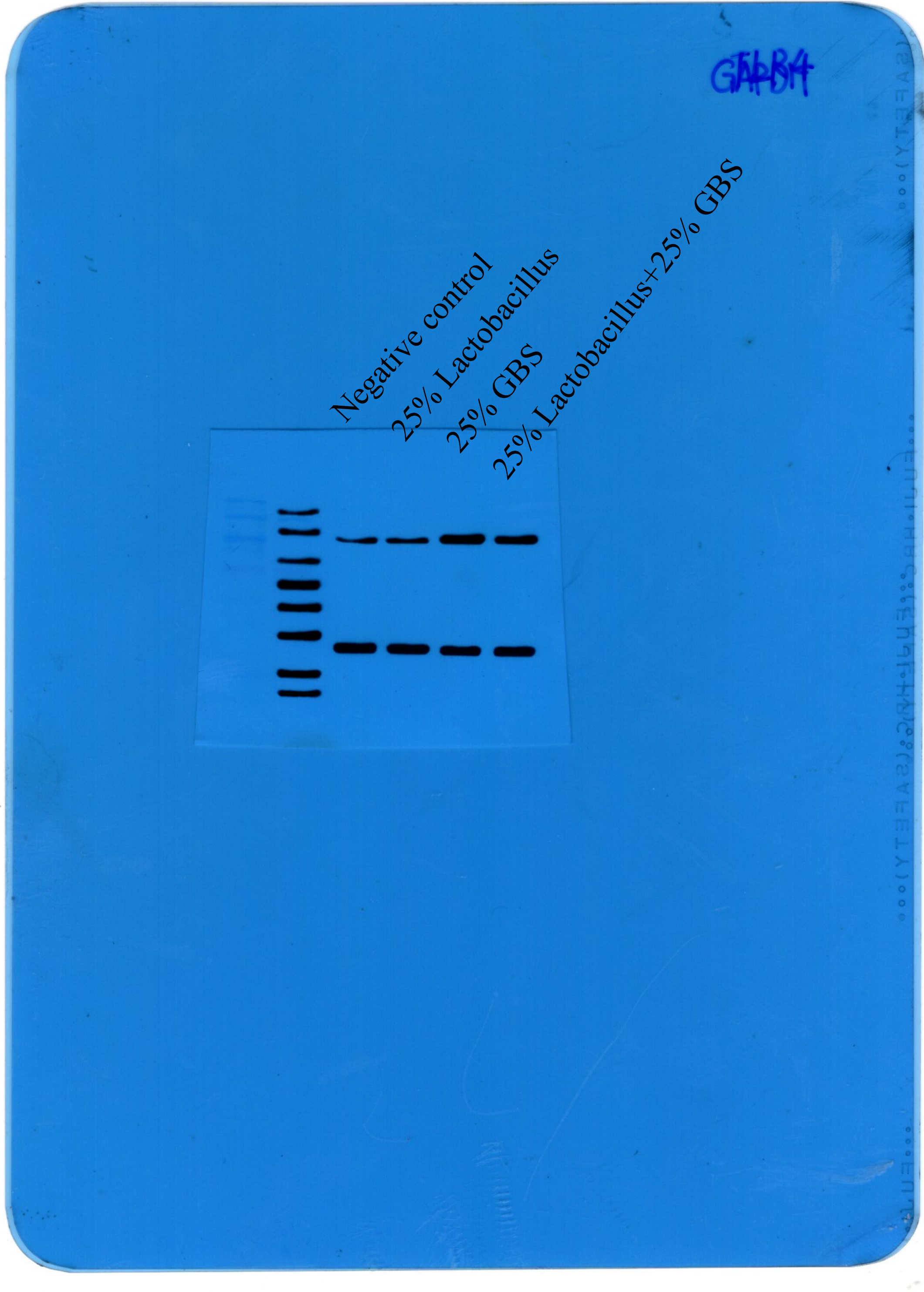

Supplement: Supplementary file 1 — Supplementary Material 1. Supplementary Fig. 1 Full-length gel/blotting image of NF-kB protein. Supplementary Fig. 2 Full-length gel/blotting image of TLR4 protein. Supplementary Fig. 3 Full-length gel/blotting image of TLR2 protein. Supplementary table 1 Gene primer information. Supplementary table 2 The fold-change values of gene. [file 12866_2025_4203_MOESM1_ESM.zip › Supplementary/Supplementary gel image TLR4.pdf]
